# FBXL4: safeguarding against mitochondrial depletion through suppression of mitophagy

**DOI:** 10.1080/15548627.2024.2318077

**Published:** 2024-02-29

**Authors:** Prajakta Kulkarni, Giang Thanh Nguyen-Dien, Keri-Lyn Kozul, Julia K. Pagan

**Affiliations:** a Faculty of Medicine, School of Biomedical Sciences, University of Queensland, Brisbane, QLD, Australia; b Department of Biotechnology, School of Biotechnology, Viet Nam National University-International University, Ho Chi Minh City, Vietnam; c Institute for Molecular Bioscience, The University of Queensland, Brisbane, QLD, Australia; d Faculty of Medicine, The University of Queensland Frazer Institute, Brisbane, QLD, Australia

**Keywords:** BNIP3, BNIP3L/NIX, FBXL4, mitophagy, MTDPS13, ubiquitin ligase

## Abstract

Mitophagy is a critical mitochondrial quality control process that selectively removes dysfunctional or excess mitochondria through the autophagy-lysosome system. The process is tightly controlled to ensure cellular and physiological homeostasis. Insufficient mitophagy can result in failure to remove damaged mitochondria and consequent cellular degeneration, but it is equally important to appropriately restrain mitophagy to prevent excessive mitochondrial depletion. Here, we discuss our recent discovery that the SKP1-CUL1-F-box (SCF)-FBXL4 (F-box and leucine-rich repeat protein 4) E3 ubiquitin ligase localizes to the mitochondrial outer membrane, where it constitutively mediates the ubiquitination and degradation of BNIP3L/NIX and BNIP3 mitophagy receptors to suppress mitophagy. The post-translational regulation of BNIP3L and BNIP3 is disrupted in mitochondrial DNA depletion syndrome 13 (MTDPS13), a multi-systemic disorder caused by mutations in the *FBXL4* gene and characterized by elevated mitophagy and mitochondrial DNA/mtDNA depletion in patient fibroblasts. Our results demonstrate that mitophagy is not solely stimulated in response to specific conditions but is instead also actively suppressed through the continuous degradation of BNIP3L and BNIP3 mediated by the SCF-FBXL4 ubiquitin ligase. Thus, cellular conditions or signaling events that prevent the FBXL4-mediated turnover of BNIP3L and BNIP3 on specific mitochondria are expected to facilitate their selective removal.

Mitophagy is induced in response to various physiological conditions and stressors, including mitochondrial damage and hypoxia. This process is initiated through various signals that mark the outer membrane of selected mitochondria, allowing the autophagy-related (ATG) machinery to identify and target those mitochondria for degradation. Extensive research has focused on understanding the mechanisms by which mitophagy is induced by mitochondrial damage. However, our understanding of how alternative mitophagy mechanisms adjust mitochondrial numbers in response to metabolic changes during differentiation or physiological stresses like hypoxia, is more limited. Under these conditions, mitophagy is induced by the upregulation of mitophagy receptors such as BNIP3 and BNIP3L, which reside in the mitochondrial outer membrane and serve to recruit the ATG machinery that mediates the *in situ* formation of autophagosomes .

When we initiated our investigations into the post-translational regulation of BNIP3L and BNIP3, it was widely accepted that the primary regulation of these proteins was at the transcriptional level via HIF1A/HIF1α, the master regulator of hypoxia-induced mitophagy. However, we speculated that additional regulatory mechanisms should exist to control the abundance of BNIP3L and BNIP3 to restrict mitophagy. Specifically, we asked whether a member of the largest family of multi-subunit E3 ligases, the CUL (cullin)-RING ligase (CRL) family, could regulate BNIP3L and BNIP3 levels. Indeed, through various techniques such as the pharmacological inhibition of CRLs, siRNAs targeting the CUL family members, the expression of dominant-negative CUL proteins, and a candidate-based siRNA screening approach, we discovered that the SCF-FBXL4 ubiquitin ligase localizes to the mitochondrial outer membrane to mediate the ubiquitination and proteasomal turnover of BNIP3L and BNIP3 [1]. FBXL4 is one of 69 interchangeable F-box proteins that act as substrate adaptors for SCF E3 ubiquitin ligase complexes.

After identifying FBXL4 as a regulator of BNIP3L and BNIP3 protein levels, our subsequent experiments assessed the overall impact of BNIP3L and BNIP3 stabilization on mitophagy. As predicted, we observed that cells lacking FBXL4 not only show an accumulation of BNIP3L and BNIP3 but also display elevated mitophagy that can be reversed by co-depletion of BNIP3L and BNIP3. To provide further validation and to investigate the specific contribution of BNIP3L and BNIP3 to mitophagy induction, we identified deletion mutants in BNIP3L and BNIP3 that are hyper-stable compared to their wild-type counterparts. When we induce the expression of either of these hyper-stable mutants, mitophagy is increased compared with cells expressing the wild-type proteins. This result suggests that the increased mitophagy observed in FBXL4-deficient cells is indeed a consequence of BNIP3L and BNIP3 stabilization, rather than stabilization of an additional, unknown FBXL4 substrate. We concluded that FBXL4 suppresses mitophagy by inducing the destabilization of BNIP3L and BNIP3 mitophagy receptors.

The mechanisms by which the stabilized mitophagy receptors coordinate with key ATG proteins to promote mitophagy remain elusive. Although FBXL4 deficiency causes the widespread accumulation of BNIP3L and BNIP3 on all mitochondria, only a fraction of these mitochondria undergo mitophagy. This implies that mitophagy might occur in a spatially restricted manner and/or that other factors determine whether mitochondria are ultimately targeted to lysosomes, for example access to a limited concentration of ATG proteins such as ULK1, or mitochondrial fission factors. Additionally, alongside mitophagy receptor stabilization, mitophagy induction might require additional “eat-me” signals on either the mitophagy receptors or elsewhere on mitochondria.

To elucidate the connection between FBXL4, and BNIP3 and BNIP3L regulation in the context of MTDPS13 we asked whether the pathogenic FBXL4 variants responsible for MTDPS13 interfere with FBXL4’s ability to suppress BNIP3 and BNIP3L levels. Indeed, we observed that patient-derived FBXL4 variants do not efficiently assemble into the holo-SCF complex, have impaired ability to mediate BNIP3L and BNIP3 turnover, and ultimately are less effective at suppressing mitophagy. We also determined that levels of BNIP3L and BNIP3 in fibroblasts derived from a patient homozygous for the p.Arg435* *FBXL4* variant contain elevated levels of mitophagy that are suppressed by co-depletion of BNIP3L and BNIP3. Thus, it appears that the mitochondrial depletion characteristic of MTDPS13 is caused by the hyperaccumulation of BNIP3L and BNIP3. Interestingly, to date, no BNIP3L or BNIP3 mutations that confer hyper-stability have been described in mitochondrial diseases.

Exploring the therapeutic potential of targeting the increased mitophagy observed in the FBXL4-associated MTDPS13 disorder, an incurable disease at present, is an area of significant interest. Therapeutic potential exists for treating patients displaying excessive mitophagy by using autophagy/mitophagy inhibitors. Consequently, it is important to determine whether mitochondrial function is restored in FBXL4-deficient patient cells after autophagy inhibition. More targeted strategies could involve the use of chemical degraders of BNIP3 and/or BNIP3L to reduce their levels.

The underlying mechanisms governing the interaction between FBXL4, and BNIP3 and BNIP3L remain to be elucidated. Post-translational modifications may play a role in either promoting or disrupting FBXL4’s recognition of BNIP3 and BNIP3L. Thus, the identification of upstream regulatory enzymes that modulate the stability of BNIP3L and BNIP3 in basal conditions, as well as the identification of post-translational modifications of FBXL4, its cofactors, and/or BNIP3 and BNIP3L, will shed light on the precise regulatory mechanisms involved. Likewise, the identification of signaling or metabolic conditions that disrupt the recognition of BNIP3L and BNIP3 by FBXL4, directly or indirectly, will be informative.

Indeed, the regulation of FBXL4 function remains largely unexplored, leaving numerous intriguing questions unanswered. Is FBXL4 abundance regulated in response to changes in conditions like metabolic state, hypoxia, iron depletion, or in specific signaling environments? Similarly, does the localization of FBXL4 or its cofactors to the outer membrane change in response to those conditions, globally or in a spatially restricted manner? Do specific tissues or developmental stages express low levels of FBXL4 protein reflecting high basal mitophagy levels and vice versa? Is FBXL4 function controlled by a cognate deubiquitinating enzyme that removes FBXL4-generated ubiquitin chains thereby stabilizing BNIP3L and BNIP3 to facilitate basal mitophagy?

In summary, our findings [1], along with complementary results published alongside ours by Sylvie Urbe’s and Hui Jiang’s research groups, provide compelling evidence suggesting that FBXL4 targets BNIP3L and BNIP3 for proteasomal degradation to ultimately restrict mitophagy, and that this mechanism is dysregulated in FBXL4-associated MTDPS13 (Figure 1). It remains to be established whether there are other clinically relevant substrates of the SCF-FBXL4 ligase complex.

**Figure 1.**
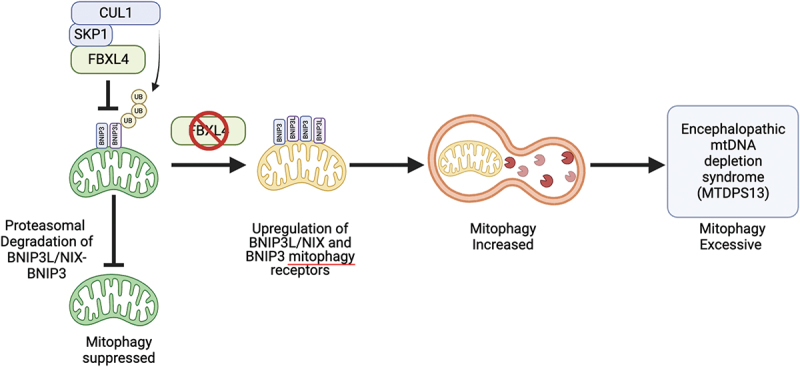
Model for SCF-FBXL4 function in mitophagy suppression. The SCF-FBXL4 ubiquitin ligase localizes to the mitochondrial outer membrane and mediates the constitutive ubiquitination and subsequent proteasomal degradation of BNIP3L and BNIP3 mitophagy receptors, thereby preventing excessive mitophagy. Loss of FBXL4 results in the stabilization and upregulation of these receptors, which ultimately leads to increased mitophagy. Pathogenic variants in FBXL4 associated with MTDPS13 are unable to effectively downregulate BNIP3L and BNIP3, leading to their accumulation and consequent elevated mitophagy. Figure was created with BioRender.
